# Structure of Bee Communities in Marginal Lands of the Puget Sound, USA

**DOI:** 10.1002/ece3.72049

**Published:** 2025-09-02

**Authors:** Evan Sugden, Will Peterman, Robert Redmond, Riley M. Anderson, David W. Crowder

**Affiliations:** ^1^ Entomo‐Logic Salt Lake City Utah USA; ^2^ Bee Search Seattle Washington USA; ^3^ The Common Acre Seattle Washington USA; ^4^ Department of Entomology Washington State University Pullman Washington USA

**Keywords:** airport, conservation, native bee, pollinators, power line corridor, urban ecosystems

## Abstract

Wild bee communities in urban ecosystems are often challenged by habitat fragmentation and low floral diversity. In such settings, marginal land surrounding airports or in power line corridors may support bees, even with small habitat patches. However, temporal surveys of wild bees are lacking for many urban areas such as the Puget Sound region of western Washington State, USA. Here, we conducted wild bee surveys at three peri‐urban sites in the Puget Sound over 7 years. Specifically, a standardized protocol was used to sample wild bee communities monthly from April to October at two sites associated with airports and one site in a power line corridor. In total, our surveys collected 25,441 specimens representing 118 confirmed species within 24 genera, with individual subsites having between 15 and 35 species in any year. The Halictidae represented the largest number of individuals collected, with 47% of specimens. By genus, *Lasioglossum* was the most speciose (*n* = 21), with *Bombus*, *Osmia*, and *Andrena* also ubiquitous and diverse. Bee diversity was high across spring and summer, and our surveys resolved the presumptive overlap of parasites with their hosts. Our study shows that marginal lands requiring little management can support diverse wild bee communities in urban areas. Our work also provides a baseline for future evaluations of wild bee communities in the Puget Sound and broader Pacific Northwest.

## Graphical abstract

We surveyed wild bee communities in marginal lands of the Puget Sound, USA. Bee communities were highly diverse and stable across seasons, but were variable within seasons.

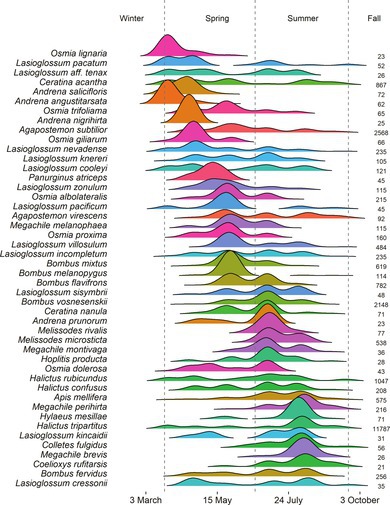

## 1 Introduction

Wild bee communities are impacted by abiotic conditions as well as the availability of nectar, pollen, and nesting and habitat resources (Plos et al. 2023). As natural habitats are increasingly lost to urbanization and agriculture, bees lose resources, which may lead to population declines (Cameron et al. 2011; Goulson et al. 2015). To aid pollinator assessment and conservation, studies are needed to establish a baseline bee community of species diversity and abundance to guide future evaluations and restoration efforts. Given that the dynamics of bee communities and their local floral resources can be highly ephemeral, capturing temporal trends within and across multiple seasons may be particularly valuable (Bloom et al. 2022, 2023).

Urban and peri‐urban habitats present unique challenges for bees due to the large amount of impervious surface, which reduces ground‐nesting, combined with extreme fragmentation of resources (Braman and Griffin 2022). Efforts to restore bee communities in urban areas have taken many forms, such as the restoration of urban lots into pollinator‐friendly spaces with diverse native flowering plants that provide continuous food resources (Poole et al. 2025). These efforts, in general terms, tend to increase bee richness and abundance (Tonietto and Larkin 2017). However, government‐leased lands, such as parks and utility corridors, can also be used for pollinator meadows, which may not only increase floral diversity but also establish nesting substrates that are key for solitary bees (Russell et al. 2018; Phillips et al. 2020; Campbell et al. 2023). Specific practices, like minimizing lawn mowing or incorporating green roofs, improve habitat quality while enhancing ecological connectivity in urban landscapes (Turo and Gardiner 2019). By leveraging city, state, or federal government‐managed land for habitat, cities could create interconnected networks of pollinator refuges, fostering urban biodiversity and ecological resilience with minimal input and management.

Efforts to enhance bee habitat in urban areas have recently focused on airports, with Chicago O'Hare, Indianapolis, and Seattle‐Tacoma Airports among those that have turned underdeveloped land into bee habitats (Lurie et al. 2022). There is often considerable land around airports (200–5000 ha) given the need for undeveloped space for aircraft, and land formerly used for airports that have been decommissioned may also provide habitat (Lurie et al. 2022). Many of these underdeveloped marginal lands may offer substantial habitat and resources to bees. Understanding how marginal lands surrounding airports compare to other habitats is key to determining if restoration efforts are needed to support diverse and robust bee communities (Braman and Griffin 2022).

Our study evaluated the diversity and structure of wild bee communities in marginal lands of western Washington State, USA. Bee diversity of western Washington has remained largely undescribed, outside of diversified farms (Bloom et al. 2019, 2022, 2023), remnant prairie (Waters et al. 2014, 2024), and montane Okanagan‐Wenatchee National Forest (Wilson et al. 2010). Given the urbanization of Western Washington, assessments are needed to determine the status of common and rare species (Bartholomew et al. 2024). To address this, we conducted monitoring over 7 years at three sites in western Washington, two at airports and one on government‐leased land in power corridors. Our focus was to document the bees in the study areas and the temporal dynamics of each species. Although this research is descriptive, we strove to answer some basic questions, including: (i) Could we approximate, in species accumulation terms, the total richness of the focal communities? (ii) How do the study sites compare in bee community structure, and can they be seen to constitute a continuous habitat? (iii) How does the structure of bee diversity in our study area compare to similar habitats in other regions? and (iv) what are the seasonal dynamics of the bee species found in western Washington?

## 2 Methods

### 2.1 Study System

Our work assessed the diversity and community structure of wild bees within and across seasons at three sites in the lower Puget Sound region in western Washington State, within a few miles of the major cities of Seattle (two sites) or Everett (one site). All sites were similar in their proximity to development, past disturbance, and largely weedy vegetation. All sites were subject to intermittent land management efforts, of which bee habitat creation was an acknowledged but secondary goal. Long‐term climatic features and respective research areas utilized are listed in Table 1. All survey sites were located in open areas with little or no tree cover. Site areas in Table 1 were calculated by drawing a perimeter around contiguous open areas within 50 m of at least one sampling station using Google Earth, and measuring the enclosed areas. By this measure, the POS site was a single contiguous area, while BPF and SCL consisted of three and four disjoint islands, respectively. Unique features of the sites are given anecdotally below.

**TABLE 1:** Site descriptions, physical features. Site abbreviations explained in the text. Calculation of the utilized area is described under Methods.

Site	Area (ha)	Ave. Ann. Ppt. (mm)	Ave. Temp. per Month (High/Avg/Low) (°C)					
			April	May	June	July	Aug.	Sept.
BPF	20.0	820	14‐10‐7	18‐12‐9	20‐15‐11	23‐17‐13	23‐18‐13	20‐15‐12
POS	4.5	990	15‐10‐7	19‐13‐9	12‐16‐12	25‐18‐14	25‐19‐14	21‐16‐12
SCL	2.5	850	16‐11‐7	19‐14‐10	22‐17‐13	25‐19‐15	25‐19‐15	22‐16‐12

*Note:* Climate data for sites BPF, POS, and SCL are taken from records at nearby airports KPAE, KSEA, and KBFI respectively. Temperature records are from the NOAA Integrated Surface Hourly data set, aggregated by weatherspark.com. Precipitation records are from the NOAA Daily Summaries data set, 1999–2024.

The Port of Seattle site (POS) is located immediately south of the airport runways of SeaTac Airport on land serving as a security buffer. The vegetation is generally weedy but varied, with patches of past revegetation, a strip of riparian corridor, and common species that include introduced wild Himalayan blackberry (*Rubus aermeniacus* Focke), introduced knapweed (*Centaurea* sp.), native and introduced wild mustard (*Brassica* spp.), and native big leaf maple ( *Acer macrophyllum* Pursh). Open areas with low perennial or annual weedy plants provided the main collecting habitat. Trap stations were separated by 50–250 m. This site was within flight range of two honey bee apiaries, each with up to 10 colonies at distances of 225 m and 2.4 km from the nearest trap stations. A total of 22 trap station positions were established in the greater area with up to 8 stations in use in any given year; utilized 2014–2020.

The Boeing Paine Field site (BPF) is 10 km southwest of Everett, WA, and spread across strips of land peripheral to industrial buildings, runways, and parking lots. Habitat included low‐maintenance lawn and weedy meadows and riparian corridors, surrounded by the edge of red alder ( *Alnus rubra* Bong.). This site is 1 km from the water of Puget Sound and experiences maritime influence, as indicated by generally cooler temperatures compared to the other sites (Table 1). A total of 8 collecting stations with separations from 50 to 750 m were utilized during 2018–2020.

The Seattle City Light site (SCL) is a power corridor 10 km southeast of Seattle. This is a discontinuous site, with 5 subsites, each containing one station and separated by 150 m to 1.9 km along an electric transfer line. Two of the five trap stations were in a semi‐managed meadow in the Rainier Beach neighborhood, one was in a semi‐boggy slope descending toward the interstate, one was in the bounds of a weedy equipment storage pad north of the Duwamish Hill Preserve, and one was on the banks of the Duwamish River between a road and a power transfer facility. These sites were used from 2014 to 2016.

### 2.2 Collecting Methodology

Trap stations consisted of linear arrays of 15, 15‐cm plastic Solo bowls, placed in 5 triplet clusters, each of which had one blue, one yellow, and one white (Figure S1). The 15‐cm bowls were replaced in 2016 with 4 oz. “minibowls”; bowl size has limited impact on abundance or richness of bees (Gonzalez et al. 2020). We also placed 3 “blue vane” traps (BanfieldBio, Woodinville, WA) at each station. Arrays that combine bee bowls and blue vane traps are widely used in bee surveys, as they are effective at capturing diverse bee species (Moreira et al. 2016; Campbell et al. 2023). However, due to the potential presence of the threatened *Bombus occidentalis* Greene at the Boeing Paine Field site, we only used one blue vane trap at this site. Traps were placed at each site once per month from April to September, with arrays placed between 08:00 and 10:00 and left for 24 h before collection. Traps were supplemented with net collecting on an opportunistic basis (Turley et al. 2024) by conducting 100 sweeps of blooming vegetation with a heavy 40 cm sweep net bag. Specimens were preserved temporarily in dry blotter paper “layers” and later rehydrated for later work. The total estimated trap effort (measured in trap days) per site across all years is shown in Figure S2.

Specimens were removed from traps by pouring the fluid through a strainer and placing bees into vials with 70% ethanol; net‐collected specimens were dispatched with ethyl acetate and placed in blotter paper “layers” for temporary storage. In the lab, specimens were removed and grouped into initial categories of genus or morphospecies and by sex. These groups were then identified as morphospecies or species under a microscope. Specimens were identified using published papers, semi‐technical guides, and Discover Life keys (see Supporting Information for a comprehensive list of all identification materials). For difficult‐to‐identify specimens, professional assistance was sought from specialists (see Acknowledgements) by sending them samples or through in‐person visits to the respective facilities. Specimens that eluded definitive species identification were assigned as unique morphospecies.

A recent analysis of diversity‐centered studies has documented the widespread lack of adequate documentation of identification resources and the infrequency of provision of adequate voucher specimens (Packer et al. 2018). We have addressed these issues by providing an exhaustive list of published resources used in identification (Supplement), listing professionals lending expertise (Acknowledgements), and by the preparation of a voucher collection, which will be under short‐term curation by the Washington Bee Atlas and will ultimately be housed at the M. T. James Entomology Collection at Washington State University, Pullman, WA.

This research began with the expectation that a large proportion of bee species we would encounter would be from difficult‐to‐identify groups (especially the Halictidae and Andrenidae), requiring substantial lethal catch to enable identification of pinned specimens. Nevertheless, we planned to avoid overcatch of specimens as recently suggested in Montero‐Castaño et al. 2022. To wit, we employed the following methodologies: (1) Protocol of brief 24‐hour trapping period (1 day) per month, (2) reduction in number of blue vane traps from 3 to 1 per trap station at BPF compared with the other 2 sites to minimize any effect on incipient *Bombus occidentalis* population, (3) annual rotation of trap station position at POS, where spatial dimensions of the overall study area allowed use of a subset of all possible station positions. While our collection efforts were not designed to track abundance over time, we observed a general pattern of increasing abundance throughout the study at all three sites. However, bee abundance did decline at the POS site in the final year.

### 2.3 Data Analysis

All analyses were performed in R v 4.2.3 (R Core Team 2023) and the *tidyverse* ecosystem (Wickham et al. 2019). Only species with confirmed identifications were included in species‐level analyses (i.e., excluding morphospecies or species identified to genus). To assess richness saturation in our sampling, we derived species accumulation curves from permutation resampling of the subsites within years for (i) all sites with net and trap collected records; (ii) all sites with trap records; (iii) trap records from Port of Seattle; (iv) trap records from Boeing Paine Field; and (v) trap records from Seattle City Light using the R package, *vegan* v 2.6–6.1 (Oksanen et al. 2024). Preliminary analyses showed a lack of saturation (curves did not reach asymptote); therefore, we elected to estimate minimum richness following Chao et al. (2009).

To compare bee community composition across collection techniques, we visualized the proportional abundance and count of unique species in each genus across all years for (i) trap and net collected records from all sites, (ii) trap records from all sites, and (iii) net records from all sites. Similarly, we compared community composition across the three sites using trap records across all years. Further, to quantitatively describe the differences in species composition across sites, we modeled a matrix of species abundance and subsite/year combinations as a function of the three sites using a permutation MANOVA with *vegan*::*adonis2* (Oksanen et al. 2024). Site location across community composition space was visualized by plotting the first two axes of a three‐dimensional NMDS ordination (stress = 0.10) with *vegan*::*metaMDS* (Faith et al. 1987).

Defining an exhaustive list of every change in species presence and abundance across the three sites is impractical. Instead, we offer a snapshot of the major differences in community composition by training a random forest model (Breiman 2001) to define the compositional differences in sites (Anderson et al. 2024). Briefly, we constructed a classification model with site as the response variable and the matrix of species abundance across subsites and years as the predictor variables. This model was tuned and refined using the *Caret* package (Kuhn 2008); however, model accuracy and output remained consistent throughout the tuning process. We used *K*‐fold cross‐validation with 10 resampling iterations, 10 complete fold sets, 999 permutations, and 55 variables at each split. We then selected the top 10th percentile of species based on variable importance score using *randomForest*::*varImp* (Liaw and Wiener 2002). Importantly, variable importance score (mean decrease in model accuracy for each species as a predictor) should not be construed as biological importance. Rare and underrepresented species may be key indicators of ecosystem function; however, they are unlikely to influence model performance in distinguishing our sample sites by species composition. Similarly, we ascribe little utility to the predictive power of our random forest model, as specimens almost always contain collection locale information anyway. Instead, this model should be considered a complementary approach to our multivariate models (NMDS) with the added benefits of quantifying the major species abundance discrepancies across sites. We report two metrics of variable importance for the top 10th percentile species: (1) mean decrease in accuracy, the average of the out‐of‐bag prediction error for each tree and the out‐of‐bag prediction error for each permutation of each variable, normalized by the standard deviation of the differences, and (2) mean decrease in Gini, the total decrease in node impurities from splitting on each variable, averaged over all trees. These metrics indicate the relative share of overall model accuracy attributed to the inclusion of each species, with larger values indicating greater contribution.

To assess the seasonal biology of bee communities at the genus and species level, we used kernel density estimation and the *ggridges* package (Wilke 2024). First, we aggregated genus and species counts over weekly intervals summed across years and sites. For genus‐level density estimation, we used biased cross‐validation to select the smoothing parameter individually for each genus (Scott 1992). As genus‐level comparisons may include many species with potentially non‐overlapping phenologies, this nonparametric smoother avoids making assumptions about the underlying distribution and allows for multimodality. For species‐level density estimation, we used Silverman's smoothing method, which assumes a Gaussian distribution.

## 3 Results

### 3.1 Bee Community Structure

Across the three sites and 7 years we collected 25,441 bee specimens. Of these, 25,017 specimens (98.3%) were identified to species, representing 118 species from 5 families and 24 genera. Of the remaining 424 specimens, 376 (1.48%) were identified only to morphospecies, representing potentially 35 additional species within the genera *Nomada*, *Osmia*, *Sphecodes*, and *Triepeolus*. The remaining 48 specimens (0.19%) were damaged and could only be identified to genus, and none of these 48 specimens represented unique genera. We describe these 118 species and their distribution across sites in the Table S1, the sex of specimens found, and indicate species or genera that represent first detections at the county or state levels (Bartholomew et al. 2024).

**FIGURE 1:**
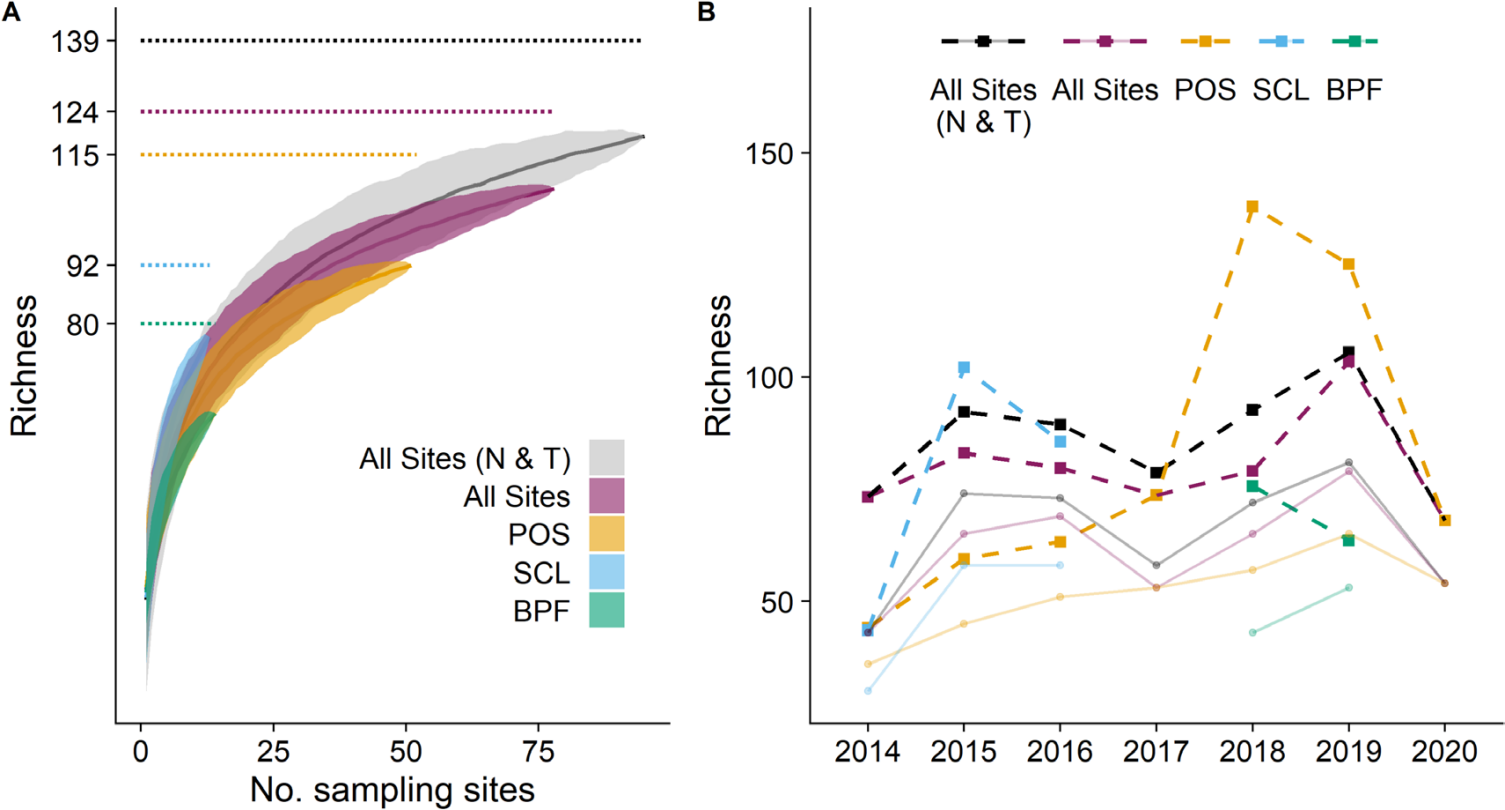
(A) Species accumulation curves for (i) all sites with traps and nets, (ii) all sites with traps only, (iii) Port of Seattle (POS); (iv) Boeing Paine Field (BPF), and (v) Seattle City Light (SCL). Curves are a function of permuted richness for each site across years by sampling effort; shaded regions are 95% confidence intervals, and dotted horizontal segments show Chao estimated richness. (B) Species richness for each site; squares and dashed lines are Chao richness for each site across years, and points and thin lines are the raw species counts for each site across years.

Our species accumulation curves did not reach asymptotes, suggesting the 118 confirmed species are likely an underestimate of the true richness across the sites (Figure 1A). Chao richness estimation suggests the minimum richness across all sites and years was 139 species (using trap and net collected records). Using only trap records, Chao richness was estimated at 124 species. Chao richness for the individual sites, Port of Seattle, Boeing Paine Field, and Seattle City Light, was estimated using trap records only at 115, 80, and 92 species, respectively. Within individual years, each site had between 15 and 40 identified species, with an estimated Chao minimum richness of between 35 and 70 species (Figure 1B). These estimates suggest that in any year, we potentially captured only between 30% and 70% of the total species richness present at each site, although we potentially captured over 85% of the total species across all years of the study. Our results also show that not all species were present every year, and only by collecting over multiple years did we gain a better estimate of the total diversity of the bee community (Figure 1).

Twenty of our species (17.0% of total) were represented by only a single specimen (Table S1). In contrast, *Halictus tripartitus* (Cockerell) had 11,787 specimens; three species had over 1000 specimens, and five more species had over 500 specimens. The ten most abundant species reflected 7 genera (Table S1). Moreover, 51 species across 15 genera were found at all three sites (Table S1). Collecting technique was non‐negligible, with 11 total species collected only by net, representing 9.3% of total richness (Table S1) and 8.7% of total specimens. As males are often underrepresented in collections, we also list species for which we collected no males (Table S1). We collected 575 *Apis mellifera* L., 2.26% of total specimens; of these, 213 were collected by net, with the remainder mostly caught in blue vane traps (Table S1).

**FIGURE 2:**
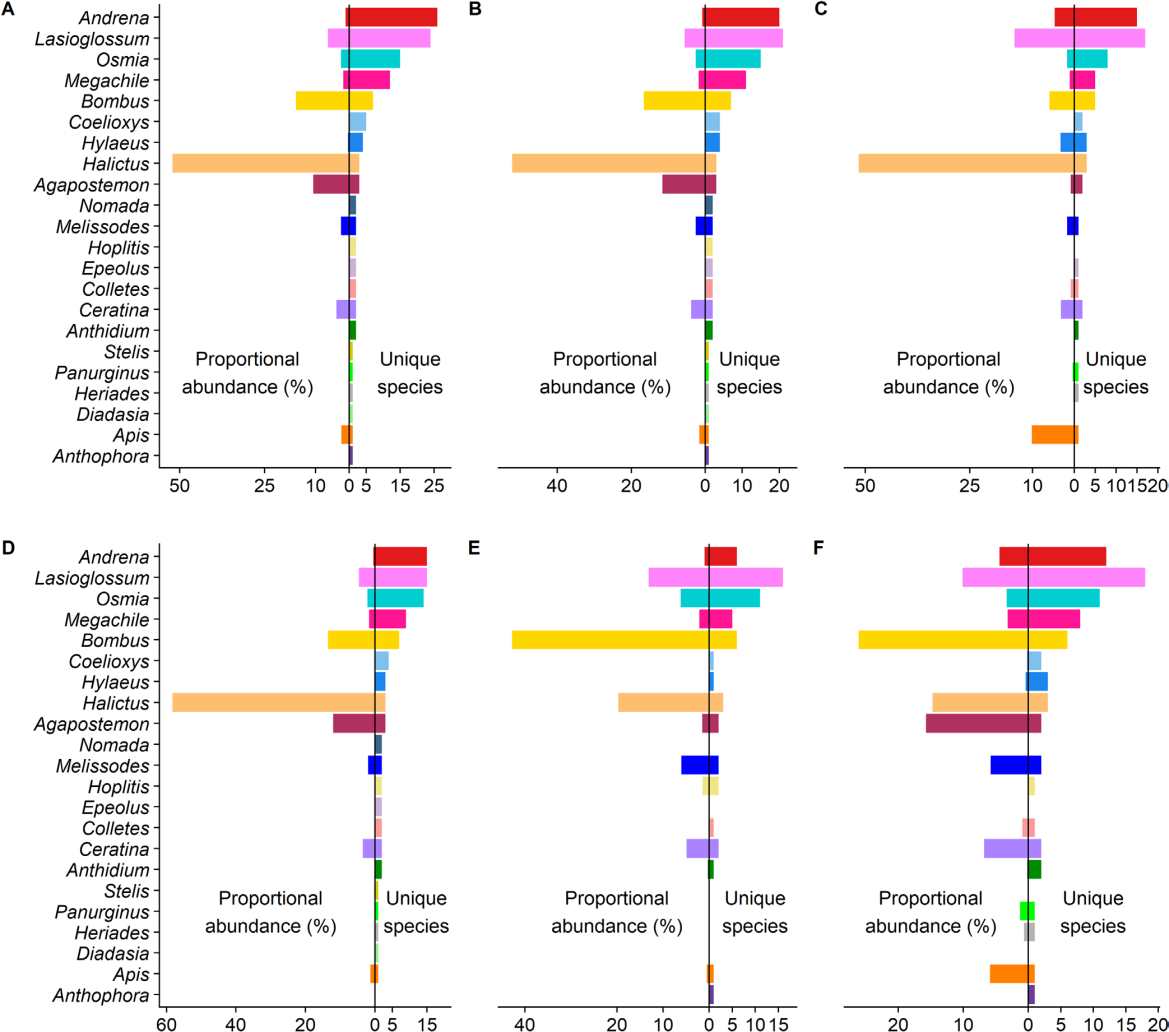
Proportional abundance and unique species for all genera, with (A) trap and net caught records from all sites and years; (B) trap records from all sites and years; (C) net records from all sites and years; (D) trap records from Port of Seattle; (E) trap records from Boeing Paine Field; and (F) trap records from Seattle City Light. The top three panels compare overall composition by collection method, while the bottom three panels compare composition by site.

Across all sites and years, the distribution of specimens across taxa was highly uneven, with 47.0% of total individuals in the genus *Halictus* (Figure 2A). Captures in traps were similar to captures in nets; except, traps appeared to capture a relatively higher proportion of *Agapostemon* and *Bombus*, while nets captured a higher proportion of *Lasioglossum* (Figure 2B,C). Overall, dominance of *Halictus* reflects greater sampling effort at the Port of Seattle, where it was most common (Figure 2D), compared to Boeing Paine Field and Seattle City Light sites, where *Bombus* was the most common taxon (42.8% and 26.1%, respectively) (Figure 2E,F). Although *Halictus* and *Bombus* were most abundant, *Lasioglossum*, *Andrena*, *Osmia*, and *Megachile* were far more speciose (Figure 2). Despite disproportionate representation of individual species between sites, diversity at the genus level was relatively conserved; a set mix of genera was typical of all sites with little variation and was even comparable between trap and net‐collected specimens (Figure 2).

**FIGURE 3:**
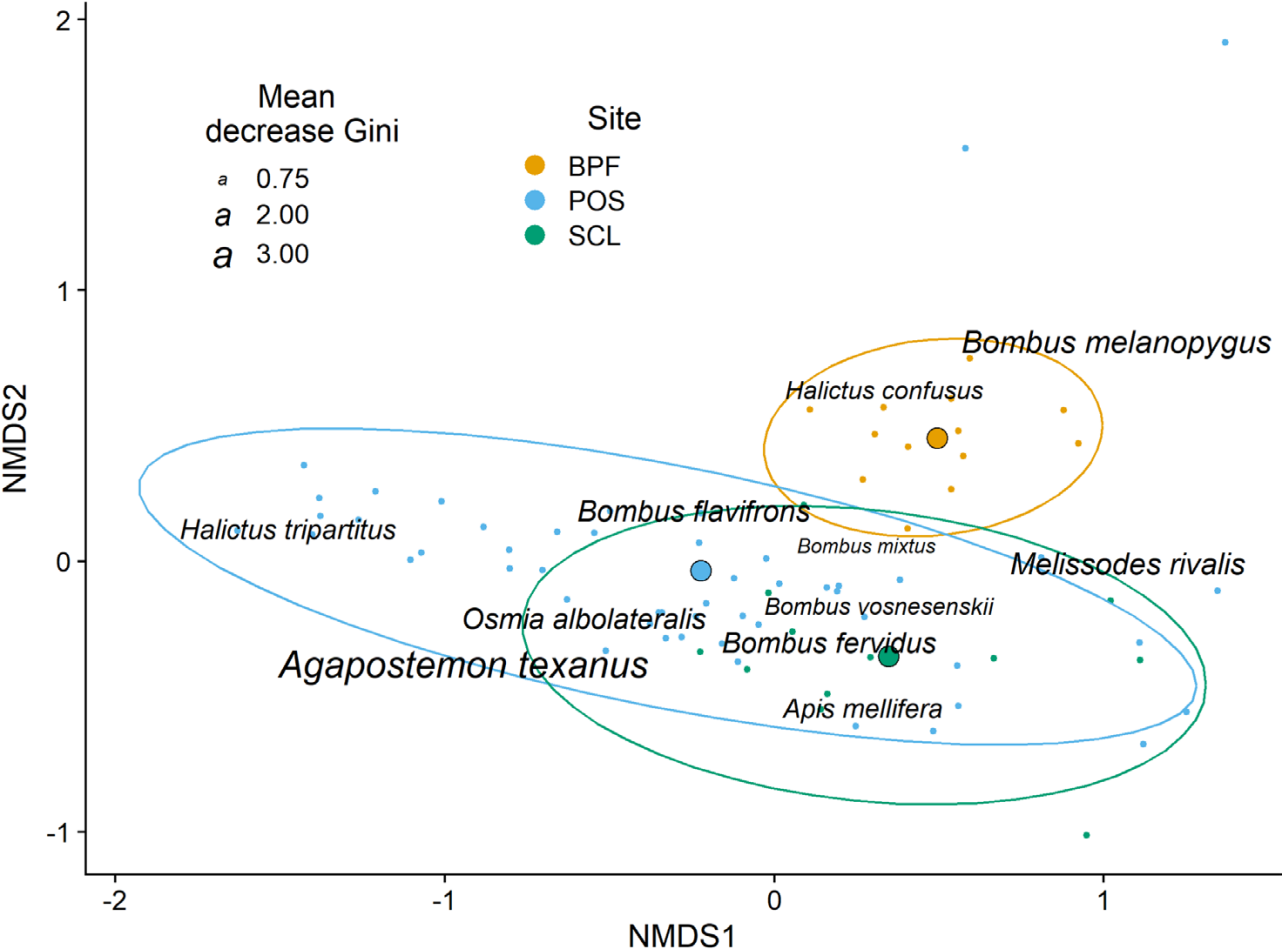
Variation in community composition across sites. Bee species are plotted on the first two axes of a three‐dimensional NMDS plot. Small points are individual subsite/year combinations of species richness and abundance. Large points are the centroids of the three sites, and ellipses are 95% confidence intervals. Bee species shown represent the top 10th percentile of compositional differences among sites. Text size is proportional to the variable importance score (Table 2).

Species composition varied significantly across sites (PERMANOVA; *F*_2_ = 2.11, *p* = 0.005), with Boeing Paine Field hosting a bee community statistically distinct from the Port of Seattle and Seattle City Light sites (Figure 3). Major changes in abundance primarily delineated the Boeing Paine Field site from the others (Table 1). Notably, when compared to the Port of Seattle and Seattle City Light sites, Boeing Paine Field displayed a relative paucity of *H. tripartitus* , *Agapostemon subtilior* (Cockerell), *A. mellifera* , and *Bombus fervidus* (Fab.), and a relative abundance in *Bombus melanopygus* Nylander, *Halictus confusus* Smith, and *Bombus mixtus* Cresson (Table 1). Moreover, our random forest model classified each site by species composition with an overall out‐of‐bag error rate of 12.8%. Error in the model is attributed to the overlap in species composition in the Port of Seattle (14.3% class error) and Seattle City Light (53.8% class error) sites. However, the Boeing Paine Field site was never misclassified (0.0% class error), further implying that the Boeing Paine Field site was quantitatively different from the others in terms of species composition. For example, the relative abundance of *Agapostemon subtilior* (Cockerell) was one order of magnitude lower in the BPF site than in the POS and SCL sites. Moreover, *Bombus melanopygus* and *Halictus confusus* were an order of magnitude more abundant in the BPF site compared to the POS and SCL sites (Table 2).

**TABLE 2:** The top 10th percentile of species ranked by variable importance score (mean decrease in Gini) from a random forest classification of site by species composition.

Species	BPF	POS	SCL	μ ↓ Accuracy	μ ↓ Gini
*Halictus tripartitus*	2.1 (38)	54.3 (11,603)	7.8 (146)	12.93	3.44
*Agapostemon subtilior*	1.3 (24)	10.6 (2269)	14.7 (275)	10.85	2.76
*Bombus melanopygus*	5 (89)	0.1 (23)	0.1 (2)	8.66	1.8
*Osmia albolateralis*	0.1 (2)	1.0 (204)	0.5 (9)	7.94	1.64
*Apis mellifera*	0.4 (8)	2.1 (444)	6.6 (123)	7.62	1.62
*Melissodes rivalis*	1.8 (32)	0.1 (17)	1.5 (28)	7.53	1.61
*Halictus confusus*	5.4 (97)	0.5 (108)	0.2 (3)	7.62	1.41
*Bombus fervidus*	0.5 (9)	0.9 (196)	2.7 (51)	6.88	1.31
*Bombus vosnesenskii*	13.9 (248)	7.3 (1556)	18.4 (344)	6.57	1.16
*Bombus mixtus*	7.6 (135)	2 (433)	2.7 (51)	2.97	0.93

*Note:* The proportional abundance (%) of each species is listed for each site, Boeing Paine Field (BPF), Port of Seattle (POS), and Seattle City Light (SCL). In parentheses are counts of each species. Mean decrease in accuracy is the permuted decrease in model accuracy by removing each species and refitting the model; mean decrease in Gini is the total decrease in node impurities from splitting each node on each species over all trees. Larger values of both indicate greater variable contribution to the model's ability to correctly classify sites by the matrix of species composition.

### 3.2 Bee Seasonal Biology

**FIGURE 4:**
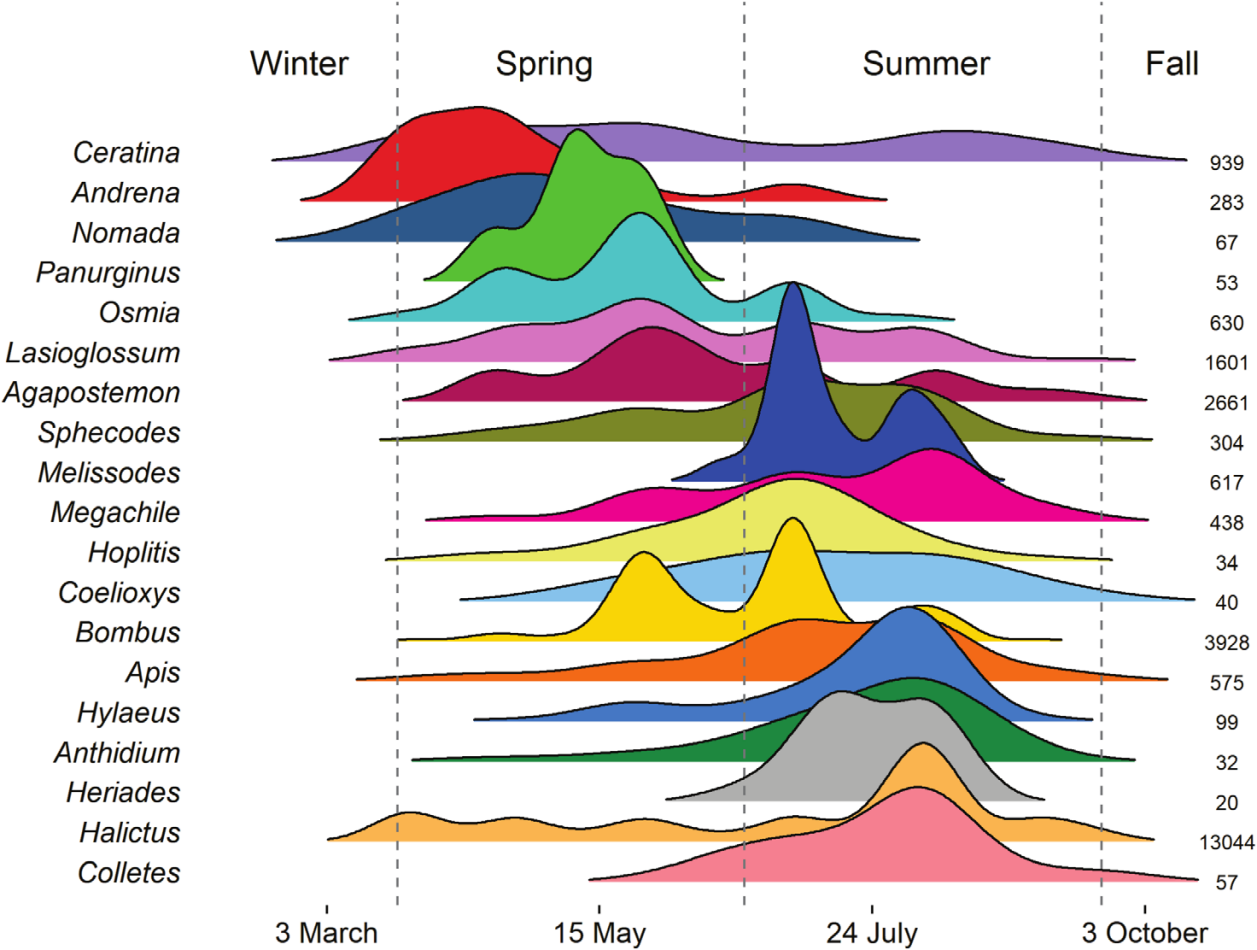
Genus‐level seasonal distributions. Sample sizes are listed, and only genera with sample sizes ≥ 20 are shown. Vertical dashed lines correspond to 21 March, 21 June, and 21 September.

**FIGURE 5:**
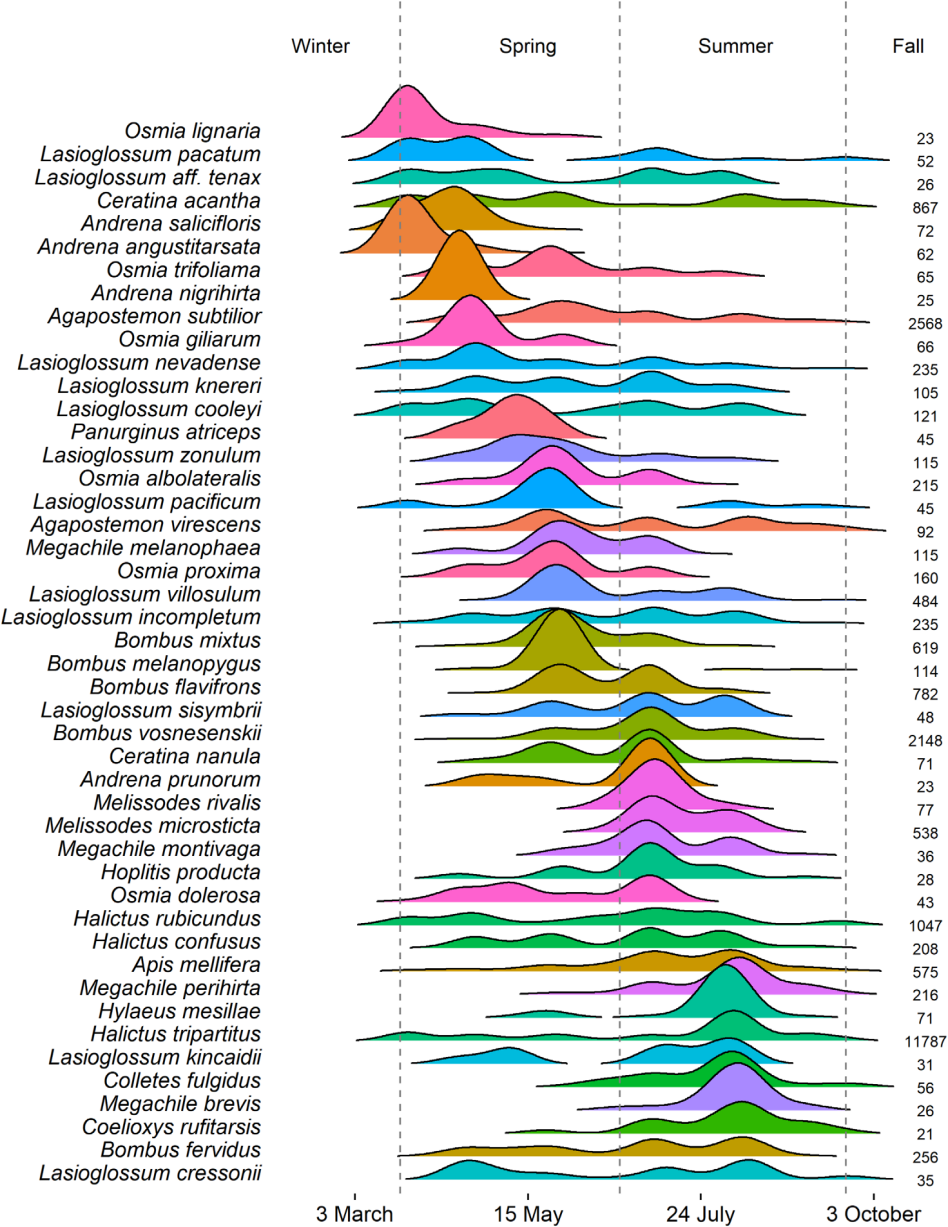
Species‐level seasonal distributions. Sample sizes are listed, and only species with sample sizes ≥ 20 are shown. Vertical dashed lines correspond to 21 March, 21 June, and 21 September.

The first bees to emerge were species in the genera *Ceratina*, *Andrena*, and *Nomada* (Figures 4 and 5). While *Andrena* and *Nomada* peaked in early spring, *Ceratina* was found throughout the entire season. The *Osmia* and *Lasioglossum* also peaked in spring with short activity periods (Figures 4 and 5). Other genera peaked in summer, including *Apis*, *Bombus*, *Halictus*, *Hylaeus*, *Megachile*, *Melissodes*, and *Sphecodes* (Figures 4 and 5). For genera, multimodal distributions likely show multiple species, as well‐defined multimodality was rare for species, with most species reaching one peak abundance before tapering off. Yet, given that major peaks in abundance are detectable despite the sampling noise suggests our data captures the true seasonal maxima. When focusing on the community level, substantial turnover occurs within seasons, but diverse communities exist across the year.

**FIGURE 6:**
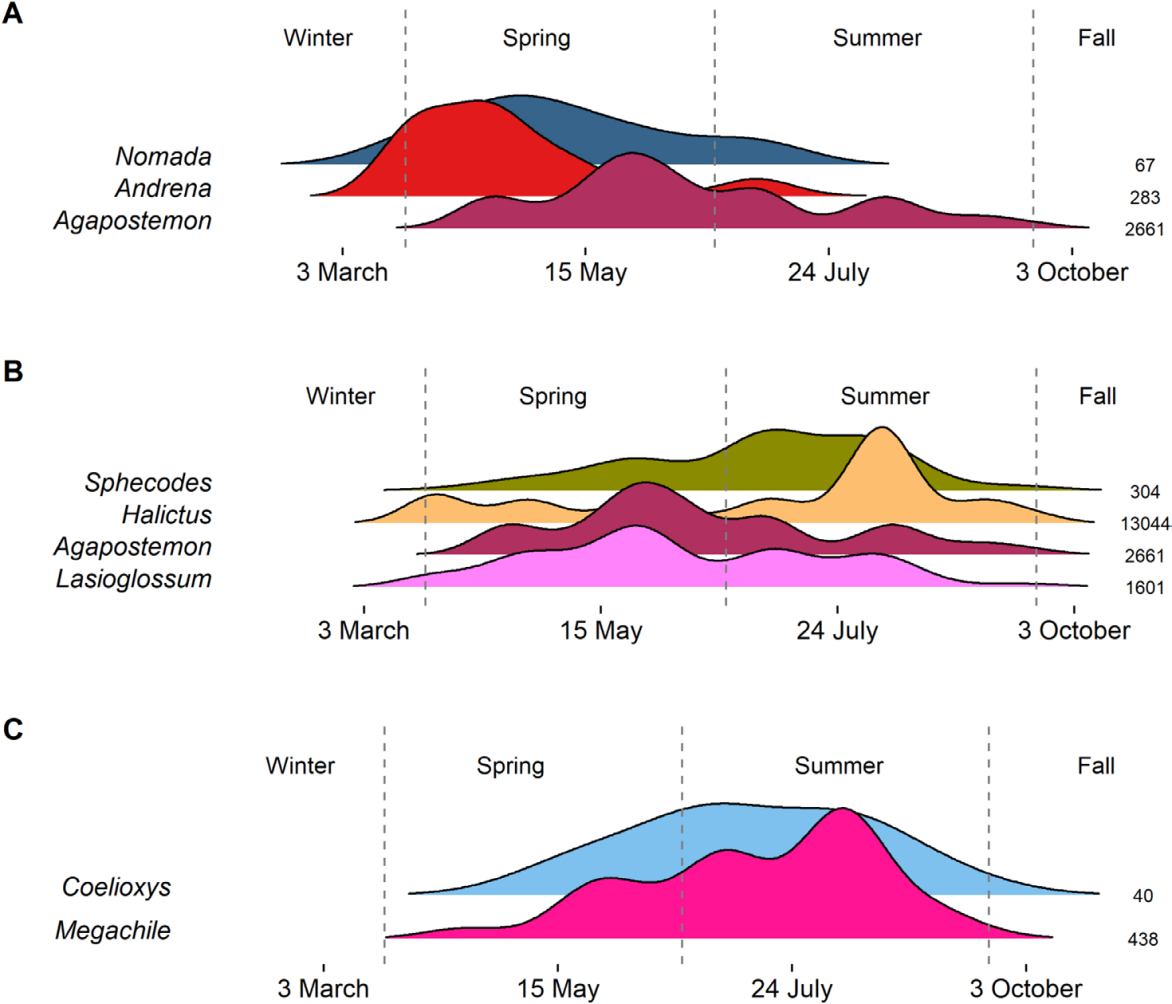
Seasonal distributions for parasites: (A) Nomada, (B) Sphecodes, and (C) Coelioxys. Beneath each parasite genus are presumed host genera. Sample sizes are the total records for each genus. Vertical dashed lines correspond to 21 March, 21 June, and 21 September.

When looking at common parasitic genera, we found major overlap in phenology with their presumed hosts (Figure 6, Figure S3). For example, *Nomada* parasites peaked in the spring at a similar time as their hosts *Andrena* and *Agapostemon* (Figure 6A), and *Coelioxys* peaked in summer at the same time as their *Megachile* hosts (Figure 6C). However, while *Sphecodes* strongly overlapped with *Halictus*, two other presumed hosts (*Agapostemon* and *Lasioglossum*) tended to be active earlier in the season (Figure 6B). Although we did not have enough specimens to reliably estimate the phenology of three other parasites (*Stelis*, *Epeolus*, *Triepeolus*), preliminary estimates of overlap between these species and their presumed hosts are shown in Figure S3.

## 4 Discussion

Our study shows that a diverse community of wild bees persists in disturbed marginal habitats of the lowland Puget Sound region of western Washington State, USA. We captured a total of 118 species across 24 genera, which exceeds the 75 species across 21 genera found in recent surveys of diversified farms (Bloom et al. 2019, 2022, 2023). Notably, our study identified an additional 35 morphospecies, suggesting we may have found up to 153 species, while Bloom et al. (2022) identified a total community of up to 109 species. Although the genera captured across both studies were largely consistent, we may have found more species with monthly sampling compared to three times a year (Bloom et al. 2022). It is also possible that the greater number of species in our sites reflected largely weedy communities compared to managed floral communities. We also note that on diversified farms and gardens Bloom et al. (2022) found 47% of their specimens were *A. mellifera* , while we found less than 3% despite the presence of active apiaries within flight distance of our collections. That such diversity was found in these intermittently managed marginal lands suggests that other disturbed or underdeveloped lands on the perimeter of more intensively developed regions may provide considerable habitat for wild bees that offers limited competition from honey bees when compared to farms.

Efforts to conserve bees in fragmented urban landscapes extend beyond airports and power lines. For example, community gardens provide a diverse range of flowering plants throughout the seasons and often support diverse pollinator communities (Turo and Gardiner 2019; Theodorou et al. 2020). Vacant lots in urban areas can also be managed to support bees by creating nesting sites and floral resources through low‐maintenance landscaping (Turo et al. 2021). These interventions not only provide forage and shelter for pollinators but also contribute to community engagement. Our study further suggests that allowing spontaneous, weedy vegetation growth in small urban areas supports pollinator diversity by offering flowering plants and nesting habitat.

Our results show diverse communities of bees were present across the season, although the composition of communities changed over time. The phenology of bee species can be impacted by many factors such as life history, climate niche, and ecological interactions. Similar to other studies, we show that bees such as *Nomada* and *Andrena* are the first to emerge in early spring, aligning with the flowering of early‐blooming plants (Moisset and Buchmann 2018). As the season progresses, the activity of genera such as *Halictus* and *Lasioglossum* becomes more prominent (Moisset and Buchmann 2018). In contrast, large‐bodied and social genera like *Apis and Bombus*, and some smaller ones, such as *Ceratina* and some species in *Halictus*, show prolonged activity, maintaining their colonies throughout the entire growing season and possibly exploiting a wider variety of floral resources. Our methodology also allowed us to resolve high‐level differences in community structure between our sites along simplified gradients. Despite the complexity, we found that communities were similar between sites both seasonally and taxonomically.

Overall, we recorded new county and state distribution records of genera, subgenera, and species (Table S1). A preponderance of such records from Boeing Paine Field may reflect the little attention Snohomish County has received from researchers. Additionally, the preponderance of *Halictus tripartitus* at the Port of Seattle and Seattle City Light deserves mention. This species is “partially eusocial” with nests that may be connected underground, a unique trait among bees (Packer et al. 2007). Several other species in the genus are known to be social and may produce large colonies (Michener 1990). In addition, despite collecting over 11,000 specimens over most seasons in all years, we never collected a male; this is like other studies that report an unusually low percentage of males in this species (Packer et al. 2007). Our populations may display the most extreme version of this trait and may be thelytokous. We suggest that sociality and perhaps thelytoky explain the extreme abundance and seasonal breadth of this species in our area.

Our study also revealed the phenology of parasitic bee groups and their hosts, although data were sparse for several taxa. Parasitic taxa such as *Nomada* and *Coelioxys* rely on phenological synchronization with their hosts to ensure access to nests and brood for parasitism. For example, *Nomada* often parasitizes *Andrena* bees, which emerge in early spring, whereas *Coelioxys* may target *Megachile* bees active during late spring and summer (Lim et al. 2022). Despite the ecological importance of these interactions, data on parasitic bees are limited. Many parasitic bees are less abundant than their hosts, and their often‐cryptic morphology, behavior, and population swings make them hard to observe (Wilson and Messinger Carril 2016). Enhanced monitoring of parasitic bees is essential to fill knowledge gaps on the roles of these key species.

Our study provides evidence that land around airports and below power line corridors can support diverse wild bee communities. These often‐underused spaces thus provide an opportunity to support biodiversity while minimally interfering with the function of the land. Airports often have extensive grassy perimeters, and restoring native plants to these areas, or reducing mowing and allowing native plants to proliferate, may create a suitable habitat for bees (Lurie et al. 2022). Power line corridors are also often cleared of non‐native vegetation, and with low maintenance can often be used to support bees (Wojcik and Buchman 2012; Russell et al. 2018). Our results corroborate findings of other studies that affix value to urban “wastelands” (Twerd and Banaszak‐Cibicka 2019). These efforts may enhance pollination services for nearby green spaces and urban farms while also promoting community development goals (Theodorou et al. 2020; Turo et al. 2021). Our results provide further evidence that marginal lands may provide a key tool for urban pollinator restoration, especially given that little pollinator‐specific maintenance on the land is required.

## Author Contributions

**Evan Sugden:** conceptualization (equal), data curation (equal), funding acquisition (equal), investigation (equal), methodology (equal), project administration (equal), writing – review and editing (equal). **Will Peterman:** conceptualization (equal), funding acquisition (equal), investigation (equal), resources (equal), writing – original draft (equal). **Robert Redmond:** conceptualization (equal), funding acquisition (equal), investigation (equal), resources (equal). **Riley M. Anderson:** data curation (equal), formal analysis (equal), investigation (equal), methodology (equal), writing – original draft (equal), writing – review and editing (equal). **David W. Crowder:** conceptualization (equal), funding acquisition (equal), project administration (equal), supervision (equal), writing – original draft (equal), writing – review and editing (equal).

## Conflicts of Interest

The authors declare no conflicts of interest.

## Supporting information


**Data S1:** ece372049‐sup‐0001‐DataS1.docx.

## Data Availability

All data and R code reported in this study have been deposited at Dryad, DOI: 10.5061/dryad.bg79cnpnx (https://doi.org/10.5061/dryad.bg79cnpnx). Ongoing developments of this work can be followed publicly at https://github.com/andersonrm/BeeSearch/tree/main.
