# Supplementary material for: Structure of Bee Communities in Marginal Lands of the Puget Sound, USA
Source: Ecol Evol. 2025 Sep 2;15(9):e72049. doi: 10.1002/ece3.72049 (PMC12404176; doi:10.1002/ece3.72049)
Supplement: Supplementary file 1 — Data S1: ece372049‐sup‐0001‐DataS1.docx. [file ECE3-15-e72049-s001.docx]

**Supporting Information for:**

Structure of Bee Communities in Marginal Lands of the Puget Sound, USA

Evan Sugden, Will Peterman, Robert Redmond, Riley M. Anderson, and David W. Crowder

**References used to Identify Bees**

All bees not identified with the literature listed below were identified and/or cross-checked in person using resources and with the help of personnel at the USDA, ARS **Pollinating Insect-Biology, Management, Systematics Research** Unit: Logan, Utah, U. S. National Pollinating Insects Collection (see Acknowledgements). Some identifications also used Discover Life keys (discoverlife.org) and were checked against reference specimens at the above facility. We received confirmatory help from other specialists as mentioned in Acknowledgements.

**References for Andrenidae, *Andrena***

Bouseman JK, LaBerge WE (1978) A revision of the bees of the genus *Andrena* of the Western Hemisphere, part IX, subgenus *Melandrena*. Transactions of the American Entomological Society 104: 275-389.

LaBerge WE (1969) A revision of the bees of the genus *Andrena* of the Western Hemisphere, part II, *Plastandrena*, *Aporandrena*, *Charitandrena*. Transactions of the American Entomological Society 95: 1-47.

LaBerge WE (1973) A revision of the bees of the genus *Andrena* of the Western Hemisphere, part VI, subgenus *Trachandrena*. Transactions of the American Entomological Society 99: 235-371.

LaBerge WE (1977) A revision of the bees of the genus *Andrena* of the Western Hemisphere, part VIII, subgenera *Thysandrena*, *Dasyandrena*, *Psammandrena*, *Rhacandrena*, *Euandrena*, *Oxyandrena*. Transactions of the American Entomological Society 103: 1-143.

LaBerge WE (1980) A revision of the bees of the genus *Andrena* of the Western Hemisphere, part X, subgenus *Andrena*. Transactions of the American Entomological Society 106: 395-525.

LaBerge WE (1985) A revision of the bees of the genus *Andrena* of the Western Hemisphere, part XI, minor subgenera and subgeneric key. Transactions of the American Entomological Society 111: 441-567.

LaBerge WE (1986) A revision of the bees of the genus Andrena of the Western Hemisphere, part XII, subgenera *Leucandrena*, *Ptilandrena*, *Scoliandrena*, and *Melandrena*. Transactions of the American Entomological Society 112: 191-248.

LaBerge WE (1989) A revision of the bees of the genus *Andrena* of the Western Hemisphere, part XIII, subgenera *Simandrena* and *Taeniandrena*. Transactions of the American Entomological Society 115: 1-56.

LaBerge WE, Bouseman JK (1970) A revision of the bees of the genus *Andrena* of the Western Hemistphere, part III, *Tylandrena*. Transactions of the American Entomological Society 96: 543-605.

LaBerge WE, Ribble DW (1972) A revision of the bees of the genus *Andrena* of the Western Hemisphere, part V, *Gonandrena*, *Geissandrena*, *Parandrena*, *Pelicandrena*. Transactions of the American Entomological Society 89: 271-358

Ribble DW (1968a) Revisions of two subgenera of *Andrena*: *Micrandrena* (Ashmead), and *Derandrena*, new subgenus (Hymenoptera: Apoidea). Bulletin of the University of Nebraska State Museum 8: 237-394.

Ribble DW (1968b) A new subgenus, *Belandrena*, of the genus *Andrena* (Hymenoptera: Apoidea) Journal of the Kansas Entomological Society 41: 220-236.

Ribble DW (1974) A revision of the bees of the genus *Andrena* of the Western Hemisphere, subgenus *Scaphandrena*. Transactions of the American Entomological Society 100: 101-189.

**References for Andrenidae, *Panurginus***

Michener CD (1935) Some Pacific Coast *Panurginus* (Hymenoptera: Apoidea). The Canadian Entomologist 67: 275-278.

**References for Apidae, *Anthophora***

Brooks RW (1983) Systematics and bionomics of Anthophora: the Bomboides group and species groups of the New World. University of California Entomology Publications 53: 436-575.

**References for Apidae, *Bombus***

Koch J, Strange J, Williams P (2012) Bumble bees of the western United States. US Dept. of Agriculture, Forest Service, Agriculture Research Service, Pollinator Partnership, pp 1-141.

Thorp RW, Horning Jr DS, Dunning LL (1983) Bumble bees and cuckoo bumble bees of California. Bulletin of the California Insect Survey 23: 1–79.

Williams P, Thorp R, Richardson L, Colla S (2014) Bumble bees of North America. Princeton University Press, Princeton, NJ, USA, 208 pp.

**References for Apidae, *Ceratina***

Daly HV (1973) Bees of the genus *Ceratina* in America north of Mexico (Hymenoptera: Apoidea). University of California Entomology Publications 74: 1-114.

**References for Apidae, *Diadasia***

Personal communication and notes from Terry Griswold and Sedonia Sipes

**References for Apidae, *Epeolus***

Onuferko TM (2018) A revision of the cleptoparasitic bee genus *Epeolus* (Latreille) for Nearctic species, north of Mexico (Hymenoptera, Apidae). Zookeys 755: 1–185.

**References for Apidae, *Melissodes***

LaBerge WE (1956a) A revision of the bees of the genus *Melissodes* in North and Central America, part I (Hymenoptera, Apidae). University of Kansas Science Bulletin 37: 911-1194.

LaBerge WE (1956b) A revision of the bees of the genus *Melissodes* in North and Central America, part II (Hymenoptera, Apidae). University of Kansas Science Bulletin 38: 533-578

LaBerge WE (1961) A revision of the bees of the genus *Melissodes* in North and Central America, part III (Hymenoptera, Apidae). University of Kansas Science Bulletin 42: 283-663

**References for Apidae, *Nomada***

Bohart GE (unpublished results) Key to males of Micronomada. USDA ARS Bee Biology and Systematics Library, Logan, UT, USA.

Bohart GE (unpublished results) Key to females of Micronomada. USDA ARS Bee Biology and Systematics Library, Logan, UT.

**References for Apidae, *Stelis***

Parker FD, Griswold TL (2013) New species of the cleptoparasitic bee genus *Stelis* (Hymenoptera: Megachilidae, Anthidiini) from the Nearctic Region. Zootaxa 3646: 529-544.

**References for Colletidae, *Colletes***

Stephen WP (1954) A revision of the bee genus *Colletes* in America North of Mexico (Hymenoptera, Colletidae). The University of Kansas Science Bulletin 36: 149-527.

**References for Colletidae, *Hylaeus***

Oram RJ (2018) Revision of the genus *Hylaeus* (Fabricius) (Hymenoptera: Colletidae) in Canada. MS Thesis at University of Regina, Regina, Saskatchewan, 165 pp.

Snelling RR (1966a) Studies on North American bees of the genus *Hylaeus*, 1: Distribution of the western species of the subgenus *Prosopis* with descriptions of new forms (Hymenoptera: Colletidae). Los Angeles County Museum Contributions in Science 98: 1-18.

Snelling RR (1966b) Studies in North American bees of the genus Hylaeus, 2: Description of a new subgenus and species (Hymenoptera: Colletidae). Proceedings of the Biological Society of Washington 79: 139-144.

Snelling RR (1970) Studies of North American bees of the genus *Hylaeus*, 5: The subgenera *Hylaeus*, s. str. and *Paraprosopis* (Hymenoptera: Colletidae). Los Angeles County Museum Contributions in Science 180: 1-60.

**References for Halictidae, *Agapostemon***

Portman ZM, Arduser M, Powley ME, Cariveau DP (2024) Taxonomy of *Agapostemon angelicus* and the *A. texanus* species complex (Hymenoptera, Halictidae) in the United States. European Journal of Taxonomy 958: 203-241.

Roberts RB (1972) Revision of the bee genus *Agapostemon* (Hymenoptera: Halictidae). University of Kansas Science Bulletin 49: 437-590.

Roberts RB (1973a) Bees of northwestern America: *Agapostemon*. Oregon State University Agricultural Experiment Station Technical Bulletin 125: 1–23.

**References for Halictidae, *Halictus***

Roberts RB (1973) Bees of northwestern America: Halictus. Oregon State University Agricultural Experiment Station Technical Bulletin 126: 1–23.

Personal communication with Doug Yanega and Joel Gardner.

**References for Halictidae, *Lasioglossum***

Gardner J, Gibbs J (2022) New and little-known Canadian *Lasioglossum* (Dialictus) (Hymenoptera: Halictidae) and an emended key to species. The Canadian Entomologist 154: 1–37.

Gibbs J (2010) Revision of the metallic species of *Lasioglossum* (Dialictus) in Canada (Hymenoptera, Halictidae, *Halictini*). Zootaxa 2591: 1-382.

McGinley RJ (1986) Studies of *Halictinae* (Apoidea: Halictidae), I: revision of New World *Lasioglossum* Curtis. Smithsonian Contributions to Zoology 429: 1-304.

**References for Megachilidae, *Anthidium***

Gonzalez VH, Griswold TL (2013) Wool carder bees of the genus *Anthidium* in the Western Hemisphere (Hymenoptera: Megachilidae): diversity, host-plant associations, phylogeny, and biogeography. Zoological Journal of the Linnean Society 168: 221-425.

**References for Megachilidae, *Coelioxys***

Baker JR (1975) Taxonomy of five Nearctic subgenera of *Coelioxys* (Hymenoptera: Megachilidae). The University of Kansas Science Bulletin 50: 649-730.

De Silva N (2012) Revision of the cleptoparasitic bee genus *Coelioxys* in Canada (Hymenoptera: Megachilidae) (Master’s Thesis). York University, York, ON, 341 pp.

**References for Megachilidae, *Heriades***

Michener CD (1938) American bees of the genus *Heriades*. Annals of the Entomological Society of America 31: 514-531.

**References for Megachilidae, *Hoplitis***

Michener CD (1947) A revision of the American species of *Hoplitis* (Hymenoptera, Megachilidae). Bulletin of the American Museum of Natural History 89: 257-318.

**References for Megachilidae, *Megachile***

Sheffield CS, Ratti C, Packer L, Griswold T (2011) Leafcutter and mason bees of the genus *Megachile* (Latreille) (Hymenoptera: Megachilidae) in Canada and Alaska. Canadian Journal of Arthropod Identification 18: 1-107.

**References for Megachilidae, *Osmia***

Sheffield CS, Ratti C, Packer L, Griswold T (2011) Leafcutter and mason bees of the genus *Megachile* (Latreille) (Hymenoptera: Megachilidae) in Canada and Alaska. Canadian Journal of Arthropod Identification 18: 1-107.

**Table S1.** Number of specimens of each species. Counts are shown for the total collection, the Port of Seatle (POS) site, the Boeing Paine Field (BPF) site, and the Seattle City Light (SCL) site. Specimens collected by trap and sweep netting, and by sex, are also reported. Species are arranged by descending abundance. The records column indicates whether species are county or state records as follows: crSNO, a species record for Snohomish County; crKING, a species record for King County; crSNO_G, a genus record for Snohomish County; crKING_G, a genus record for King County; sr, a statewide species record; srG, a statewide genus record; srSG, a statewide subgenus record. Species with blank records have been detected before. † Species found at all three sites, ◊ Species collected only by net, § Only females of species collected.

| **ID#** | **Species** | **Records** | **Total** | **POS** | **BPF** | **SCL** | **Trap** | **Net** | **Female** | **Male** |
| --- | --- | --- | --- | --- | --- | --- | --- | --- | --- | --- |
| 1 | *Halictus tripartitus*  (Cockerell, 1895) †§ | crSNO | 11787 | 11603 | 38 | 146 | 10831 | 956 | 11787 | 0 |
| 2 | *Agapostemon subtilior*  (Cockerell, 1898) † | crSNO | 2568 | 2269 | 24 | 275 | 2554 | 14 | 2420 | 148 |
| 3 | *Bombus vosnesenskii*  (Radoszkowski, 1862) † |  | 2148 | 1556 | 248 | 344 | 2102 | 46 | 2048 | 100 |
| 4 | *Halictus rubicundus*  (Christ, 1791) † |  | 1047 | 666 | 220 | 161 | 936 | 111 | 936 | 111 |
| 5 | *Ceratina acantha*  (Provancher, 1895) † | crSNO_G | 867 | 670 | 78 | 119 | 805 | 62 | 658 | 209 |
| 6 | *Bombus flavifrons*  (Cresson, 1863) † |  | 782 | 523 | 242 | 17 | 755 | 27 | 556 | 226 |
| 7 | *Bombus mixtus*  (Cresson, 1878) † |  | 619 | 433 | 135 | 51 | 572 | 47 | 532 | 87 |
| 8 | *Apis mellifera*  (Linnaeus, 1758) † |  | 575 | 444 | 8 | 123 | 362 | 213 | 571 | 4 |
| 9 | *Melissodes microstictus*  (Cockerell, 1905) † | crSNO_G | 538 | 389 | 67 | 82 | 503 | 35 | 379 | 159 |
| 10 | *Lasioglossum villosulum*  (Kirby, 1802) † |  | 484 | 348 | 98 | 38 | 307 | 177 | 414 | 70 |
| 11 | *Bombus fervidus*  (Fabricius, 1798) † |  | 256 | 196 | 9 | 51 | 253 | 3 | 202 | 54 |
| 12 | *Lasioglossum incompletum*  (Crawford, 1907) † | crKING : crSNO | 235 | 176 | 2 | 57 | 210 | 25 | 211 | 24 |
| 13 | *Lasioglossum nevadense*  (Crawford, 1907) † | crKING : crSNO | 235 | 174 | 52 | 9 | 208 | 27 | 227 | 8 |
| 14 | *Megachile perihirta*  (Cockerell, 1898) † |  | 216 | 169 | 25 | 22 | 203 | 13 | 173 | 43 |
| 15 | *Osmia albolateralis*  (Cockerell, 1906) † | crKING : crSNO | 215 | 204 | 2 | 9 | 203 | 12 | 133 | 82 |
| 16 | *Halictus confusus*  (Smith, 1853) † | crSNO | 208 | 108 | 97 | 3 | 191 | 17 | 143 | 65 |
| 17 | *Osmia proxima*  (Cresson, 1864) † | crKING : crSNO | 160 | 117 | 30 | 13 | 150 | 10 | 152 | 8 |
| 18 | *Lasioglossum cooleyi*  (Crawford, 1906) † | crKING : crSNO | 121 | 89 | 17 | 15 | 116 | 5 | 118 | 3 |
| 19 | *Lasioglossum zonulum*  (Smith, 1848) † | crSNO | 115 | 86 | 15 | 14 | 113 | 2 | 112 | 3 |
| 20 | *Megachile melanophaea*  (Smith, 1853) † | crSNO | 115 | 100 | 4 | 11 | 115 | 0 | 88 | 27 |
| 21 | *Bombus melanopygus*  (Nylander, 1848) † |  | 114 | 23 | 89 | 2 | 114 | 0 | 73 | 41 |
| 22 | *Lasioglossum knereri*  (Gibbs, 2010) † | crSNO | 105 | 77 | 22 | 6 | 95 | 10 | 83 | 22 |
| 23 | *Agapostemon virescens*  (Fabricius, 1775) † |  | 92 | 88 | 1 | 3 | 88 | 4 | 67 | 25 |
| 24 | *Melissodes rivalis*  (Cresson, 1872) † | crSNO_G | 77 | 17 | 32 | 28 | 77 | 0 | 29 | 48 |
| 25 | *Andrena salicifloris*  (Cockerell, 1897) † |  | 72 | 28 | 21 | 23 | 54 | 18 | 19 | 53 |
| 26 | *Ceratina nanula*  (Cockerell, 1897) † | crKING : crSNO_G | 71 | 64 | 5 | 2 | 66 | 5 | 55 | 16 |
| 27 | *Hylaeus mesillae*  (Cockerell, 1896) | crKING | 71 | 69 | 0 | 2 | 6 | 65 | 36 | 35 |
| 28 | *Osmia giliarum*  (Cockerell, 1906) † | crSNO | 66 | 28 | 22 | 16 | 65 | 1 | 3 | 63 |
| 29 | *Osmia trifoliama*  (Sandhouse, 1939) † | crKING : crSNO | 65 | 48 | 16 | 1 | 58 | 7 | 48 | 17 |
| 30 | *Andrena angustitarsata*  (Viereck, 1904) † | crSNO | 62 | 6 | 44 | 12 | 22 | 40 | 13 | 49 |
| 31 | *Colletes fulgidus*  (Swenk, 1904) † | crKING : crSNO_G | 56 | 38 | 1 | 17 | 38 | 18 | 48 | 8 |
| 32 | *Lasioglossum pacatum*  (Sandhouse, 1924) †§ | crKING : crSNO | 52 | 36 | 9 | 7 | 51 | 1 | 52 | 0 |
| 33 | *Lasioglossum sisymbrii*  (Cockerell, 1895) |  | 48 | 46 | 0 | 2 | 27 | 21 | 34 | 14 |
| 34 | *Lasioglossum pacificum*  (Cockerell, 1898) † | crSNO | 45 | 42 | 1 | 2 | 33 | 12 | 41 | 4 |
| 35 | *Panurginus atriceps*  (Cresson, 1878) | crSNO_G | 45 | 23 | 0 | 22 | 37 | 8 | 29 | 16 |
| 36 | *Osmia dolerosa*  (Sandhouse, 1939)† | crSNO | 43 | 39 | 1 | 3 | 42 | 1 | 34 | 9 |
| 37 | *Megachile montivaga*  (Cresson, 1878) † | crKING : crSNO | 36 | 27 | 3 | 6 | 36 | 0 | 31 | 5 |
| 38 | *Lasioglossum cressonii*  (Robertson, 1890) † |  | 35 | 21 | 3 | 11 | 34 | 1 | 33 | 2 |
| 39 | *Lasioglossum kincaidii*  (Cockerell, 1898) †§ | crSNO | 31 | 28 | 1 | 2 | 31 | 0 | 31 | 0 |
| 40 | *Hoplitis producta*  (Cresson, 1864) † | crKING_G : crSNO_G | 28 | 6 | 20 | 2 | 28 | 0 | 13 | 15 |
| 41 | *Lasioglossum tenax*  (Sandhouse, 1924) † | crKING : crSNO | 26 | 17 | 7 | 2 | 20 | 6 | 24 | 2 |
| 42 | *Megachile brevis*  (Say, 1837) | crKING | 26 | 22 | 0 | 4 | 23 | 3 | 20 | 6 |
| 43 | *Andrena nigrihirta*  (Ashmead, 1890) † | crSNO | 25 | 1 | 1 | 23 | 25 | 0 | 1 | 24 |
| 44 | *Andrena prunorum*  (Cockerell, 1896) † |  | 23 | 20 | 2 | 1 | 18 | 5 | 20 | 3 |
| 45 | *Osmia lignaria*  (Say, 1837) † |  | 23 | 2 | 10 | 11 | 23 | 0 | 16 | 7 |
| 46 | *Coelioxys rufitarsis*  (Smith, 1854) † | crSNO_G | 21 | 17 | 1 | 3 | 20 | 1 | 7 | 14 |
| 47 | *Hylaeus modestus*  (Say, 1837) † | crKING : crSNO_G | 20 | 13 | 2 | 5 | 19 | 1 | 19 | 1 |
| 48 | *Anthidium oblongatum*  (Illiger, 1806) |  | 19 | 16 | 0 | 3 | 16 | 3 | 14 | 5 |
| 49 | *Heriades carinata*  (Cresson, 1864) |  | 19 | 3 | 0 | 16 | 15 | 4 | 17 | 2 |
| 50 | *Andrena candida*  (Smith, 1879) † | crSNO | 18 | 9 | 4 | 5 | 17 | 1 | 13 | 5 |
| 51 | *Megachile angelarum*  (Cockerell, 1902) |  | 15 | 8 | 0 | 7 | 11 | 4 | 5 | 10 |
| 52 | *Lasioglossum laevissimum*  (Smith, 1853) † |  | 14 | 9 | 2 | 3 | 6 | 8 | 10 | 4 |
| 53 | *Osmia pusilla*  (Cresson, 1864) † | crKING : crSNO | 14 | 6 | 7 | 1 | 13 | 1 | 6 | 8 |
| 54 | *Anthidium manicatum*  (Linnaeus, 1758) † |  | 13 | 7 | 5 | 1 | 13 | 0 | 9 | 4 |
| 55 | *Megachile gemula*  (Cresson, 1878) | crKING | 12 | 9 | 0 | 3 | 12 | 0 | 5 | 7 |
| 56 | *Osmia pinorum*  (Cockerell, 1935) | crKING : crSNO : sr | 12 | 1 | 11 | 0 | 12 | 0 | 0 | 12 |
| 57 | *Andrena scurra*  (Viereck, 1904) ◊ | crKING | 11 | 11 | 0 | 0 | 0 | 11 | 5 | 6 |
| 58 | *Andrena hemileuca*  (Viereck, 1904) |  | 10 | 3 | 0 | 7 | 9 | 1 | 1 | 9 |
| 59 | *Coelioxys sodalis*  (Cresson, 1878) | crKING | 9 | 9 | 0 | 0 | 9 | 0 | 3 | 6 |
| 60 | *Lasioglossum buccale*  (Pérez, 1903) † | crKING : crSNO | 8 | 4 | 1 | 3 | 8 | 0 | 7 | 1 |
| 61 | *Lasioglossum inconditum*  (Cockerell, 1916) | crSNO | 8 | 1 | 7 | 0 | 8 | 0 | 7 | 1 |
| 62 | *Osmia tristella*  (Cockerell, 1897) † | crSNO | 8 | 6 | 1 | 1 | 6 | 2 | 6 | 2 |
| 63 | *Lasioglossum zephyrum*  (Smith, 1853) § | crKING | 7 | 0 | 0 | 7 | 7 | 0 | 7 | 0 |
| 64 | *Megachile rotundata*  (Fabricius, 1787) | crSNO | 7 | 0 | 2 | 5 | 7 | 0 | 3 | 4 |
| 65 | *Andrena piperi*  (Viereck, 1904) ◊§ | crKING | 6 | 6 | 0 | 0 | 0 | 6 | 6 | 0 |
| 66 | *Bombus sitkensis*  (Nylander, 1848) † |  | 6 | 1 | 3 | 2 | 6 | 0 | 2 | 4 |
| 67 | *Coelioxys porterae*  (Cockerell, 1900) | crKING : sr | 6 | 5 | 0 | 1 | 6 | 0 | 1 | 5 |
| 68 | *Epeolus compactus*  (Cresson, 1878) |  | 6 | 5 | 0 | 1 | 4 | 2 | 0 | 6 |
| 69 | *Hoplitis albifrons*  (Kirby, 1837) § | crKING_G : crSNO_G | 6 | 3 | 3 | 0 | 6 | 0 | 6 | 0 |
| 70 | *Lasioglossum ruidosense*  (Cockerell, 1897) †§ | crKING : crSNO | 6 | 1 | 4 | 1 | 5 | 1 | 6 | 0 |
| 71 | *Andrena frigida*  (Smith, 1853) |  | 5 | 5 | 0 | 0 | 4 | 1 | 2 | 3 |
| 72 | *Andrena knuthiana*  (Cockerell, 1901) § |  | 5 | 5 | 0 | 0 | 3 | 2 | 5 | 0 |
| 73 | *Andrena nigrocaerulea*  (Cockerell, 1897) § |  | 5 | 5 | 0 | 0 | 5 | 0 | 5 | 0 |
| 74 | *Andrena pallidifovea*  (Viereck, 1904) ◊§ | crKING | 5 | 5 | 0 | 0 | 0 | 5 | 5 | 0 |
| 75 | *Andrena vicina*  (Smith, 1853) ◊ |  | 5 | 5 | 0 | 0 | 0 | 5 | 2 | 3 |
| 76 | *Andrena thaspii*  (Graenicher, 1903) § |  | 4 | 3 | 1 | 0 | 3 | 1 | 4 | 0 |
| 77 | *Hylaeus punctatus*  (Brullé, 1832) § | crKING | 4 | 3 | 0 | 1 | 1 | 3 | 4 | 0 |
| 78 | *Megachile lapponica*  (Thomson, 1872) | crKING | 4 | 0 | 0 | 4 | 3 | 1 | 1 | 3 |
| 79 | *Osmia coloradensis*  (Cresson, 1878) |  | 4 | 1 | 0 | 3 | 4 | 0 | 2 | 2 |
| 80 | *Stelis subcaerulea*  (Cresson, 1878) | crKING | 4 | 4 | 0 | 0 | 4 | 0 | 0 | 4 |
| 81 | *Andrena perplexa*  (Smith, 1853) | crKING | 3 | 2 | 0 | 1 | 3 | 0 | 2 | 1 |
| 82 | *Andrena rufosignata*  (Cockerell, 1902) |  | 3 | 3 | 0 | 0 | 3 | 0 | 2 | 1 |
| 83 | *Coelioxys octodentatus*  (Say, 1824) § | crKING | 3 | 3 | 0 | 0 | 3 | 0 | 3 | 0 |
| 84 | *Lasioglossum cordleyi*  (Crawford, 1906) § | crKING : crSNO | 3 | 1 | 2 | 0 | 2 | 1 | 3 | 0 |
| 85 | *Nomada suavis*  (Cresson, 1878) | crKING | 3 | 3 | 0 | 0 | 3 | 0 | 1 | 2 |
| 86 | *Osmia nanula*  (Cockerell, 1897) § |  | 3 | 2 | 0 | 1 | 3 | 0 | 3 | 0 |
| 87 | *Osmia texana*  (Cresson, 1872) § | crKING | 3 | 2 | 0 | 1 | 2 | 1 | 3 | 0 |
| 88 | *Andrena crataegi*  (Robertson, 1893) § |  | 2 | 2 | 0 | 0 | 1 | 1 | 2 | 0 |
| 89 | *Andrena trevoris*  (Cockerell, 1897) | crSNO | 2 | 0 | 1 | 1 | 2 | 0 | 1 | 1 |
| 90 | *Andrena vicinoides*  (Viereck, 1904) |  | 2 | 0 | 0 | 2 | 2 | 0 | 0 | 2 |
| 91 | *Anthophora terminalis*  (Cresson, 1869) | crKING : crSNO_G | 2 | 0 | 1 | 1 | 2 | 0 | 1 | 1 |
| 92 | *Bombus rufocinctus*  (Cresson, 1863) § | crKING | 2 | 2 | 0 | 0 | 1 | 1 | 2 | 0 |
| 93 | *Diadasia enavata*  (Cresson, 1872) § | crKING : srG | 2 | 2 | 0 | 0 | 2 | 0 | 2 | 0 |
| 94 | *Epeolus olympiellus*  (Cockerell, 1904) | crKING | 2 | 2 | 0 | 0 | 2 | 0 | 1 | 1 |
| 95 | *Lasioglossum titusi*  (Crawford, 1902) ◊§ | crKING | 2 | 2 | 0 | 0 | 0 | 2 | 2 | 0 |
| 96 | *Megachile frigida*  (Smith, 1853) | crSNO | 2 | 1 | 1 | 0 | 2 | 0 | 1 | 1 |
| 97 | *Osmia bucephala*  (Cresson, 1864) | crSNO | 2 | 1 | 1 | 0 | 2 | 0 | 1 | 1 |
| 98 | *Osmia caerulescens*  (Linnaeus, 1758) | crSNO | 2 | 1 | 1 | 0 | 2 | 0 | 1 | 1 |
| 99 | *Agapostemon femoratus*  (Crawford, 1901) § | crKING | 1 | 1 | 0 | 0 | 1 | 0 | 1 | 0 |
| 100 | *Andrena buckelli*  (Viereck, 1924) | crKING | 1 | 0 | 0 | 1 | 1 | 0 | 0 | 1 |
| 101 | *Andrena gordoni*  (Ribble, 1974) ◊§ | crKING | 1 | 1 | 0 | 0 | 0 | 1 | 1 | 0 |
| 102 | *Andrena hippotes*  (Robertson, 1895) § | crKING | 1 | 0 | 0 | 1 | 1 | 0 | 1 | 0 |
| 103 | *Andrena miranda*  (Smith, 1879) § |  | 1 | 1 | 0 | 0 | 1 | 0 | 1 | 0 |
| 104 | *Andrena subaustralis*  (Cockerell, 1898) | crKING | 1 | 1 | 0 | 0 | 1 | 0 | 0 | 1 |
| 105 | *Andrena subtilis*  (Smith, 1879) ◊§ |  | 1 | 1 | 0 | 0 | 0 | 1 | 1 | 0 |
| 106 | *Andrena transnigra*  (Viereck, 1904) |  | 1 | 0 | 0 | 1 | 1 | 0 | 0 | 1 |
| 107 | *Coelioxys gilensis*  (Cockerell, 1898) ◊§ | crKING : srSG | 1 | 1 | 0 | 0 | 0 | 1 | 1 | 0 |
| 108 | *Colletes kincaidii*  (Cockerell, 1898) | crKING | 1 | 1 | 0 | 0 | 1 | 0 | 0 | 1 |
| 109 | *Lasioglossum nigroviride*  (Graenicher, 1911) | crSNO | 1 | 0 | 1 | 0 | 1 | 0 | 0 | 1 |
| 110 | *Lasioglossum ovaliceps*  (Cockerell, 1898) § |  | 1 | 0 | 0 | 1 | 1 | 0 | 1 | 0 |
| 111 | *Lasioglossum punctatoventre*  (Crawford, 1907) ◊§ | crKING | 1 | 1 | 0 | 0 | 0 | 1 | 1 | 0 |
| 112 | *Lasioglossum sedi*  (Sandhouse, 1924) ◊§ |  | 1 | 1 | 0 | 0 | 0 | 1 | 1 | 0 |
| 113 | *Lasioglossum yukonae*  (Gibbs, 2010) § | crSNO : sr | 1 | 0 | 0 | 1 | 1 | 0 | 1 | 0 |
| 114 | *Megachile fidelis*  (Cresson, 1878) ◊§ |  | 1 | 1 | 0 | 0 | 0 | 1 | 1 | 0 |
| 115 | *Megachile gravita*  (Mitchell, 1934) | crKING | 1 | 1 | 0 | 0 | 1 | 0 | 0 | 1 |
| 116 | *Megachile onobrychidis*  (Cockerell, 1908) § | crKING | 1 | 1 | 0 | 0 | 1 | 0 | 1 | 0 |
| 117 | *Nomada formula*  (Viereck, 1903) § | crSNO : sr | 1 | 1 | 0 | 0 | 1 | 0 | 1 | 0 |
| 118 | *Osmia densa*  (Cresson, 1864) § |  | 1 | 0 | 0 | 1 | 1 | 0 | 1 | 0 |

**Fig. S1**. Diagrammatic view of typical trap station. BVT = Blue Vane Trap. *Not to scale*.

**
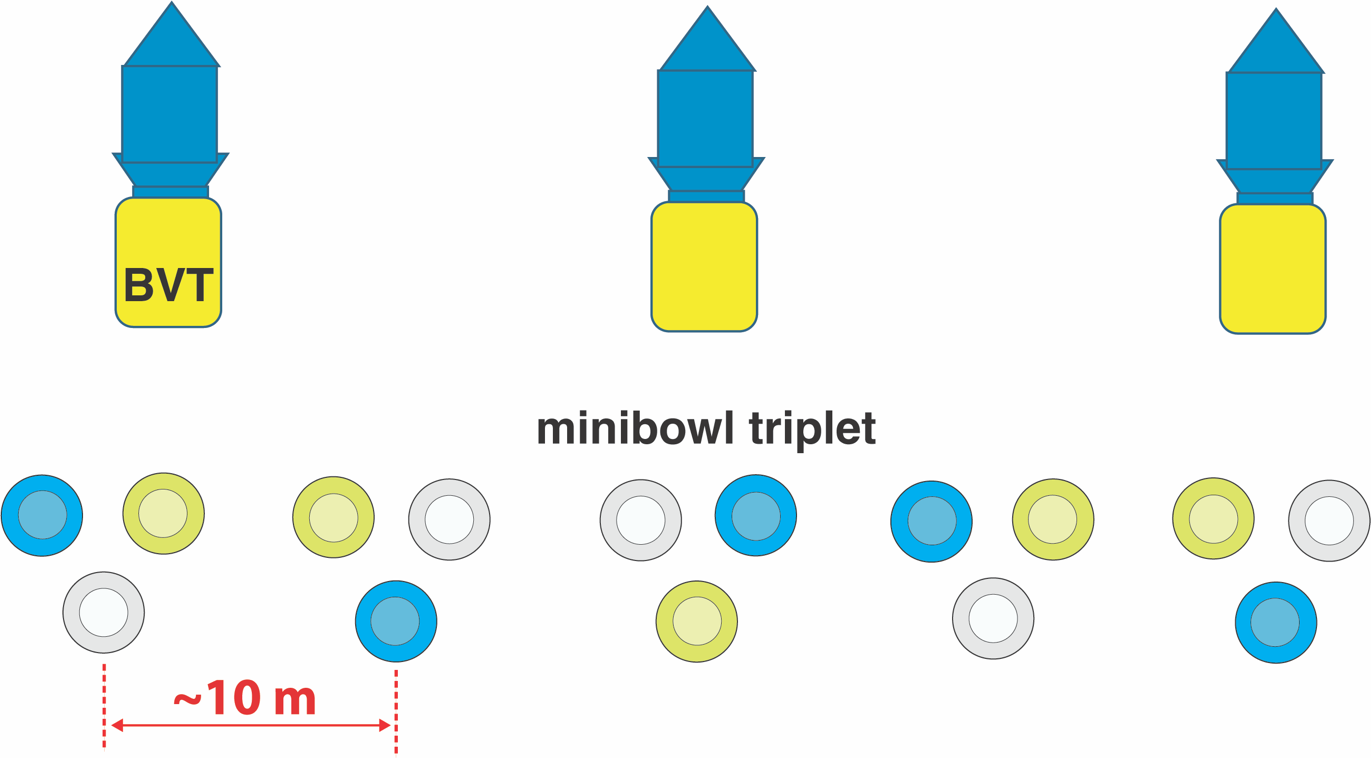
**

**Fig. S2**. Collecting effort across years at all sites. a) Trap days expressed as a standard trap set over a 24-hour period. (BPF values expressed as equivalents with reference to numbers of specimens collected by standard trap sets at other sites.) b) Net collecting events.

**
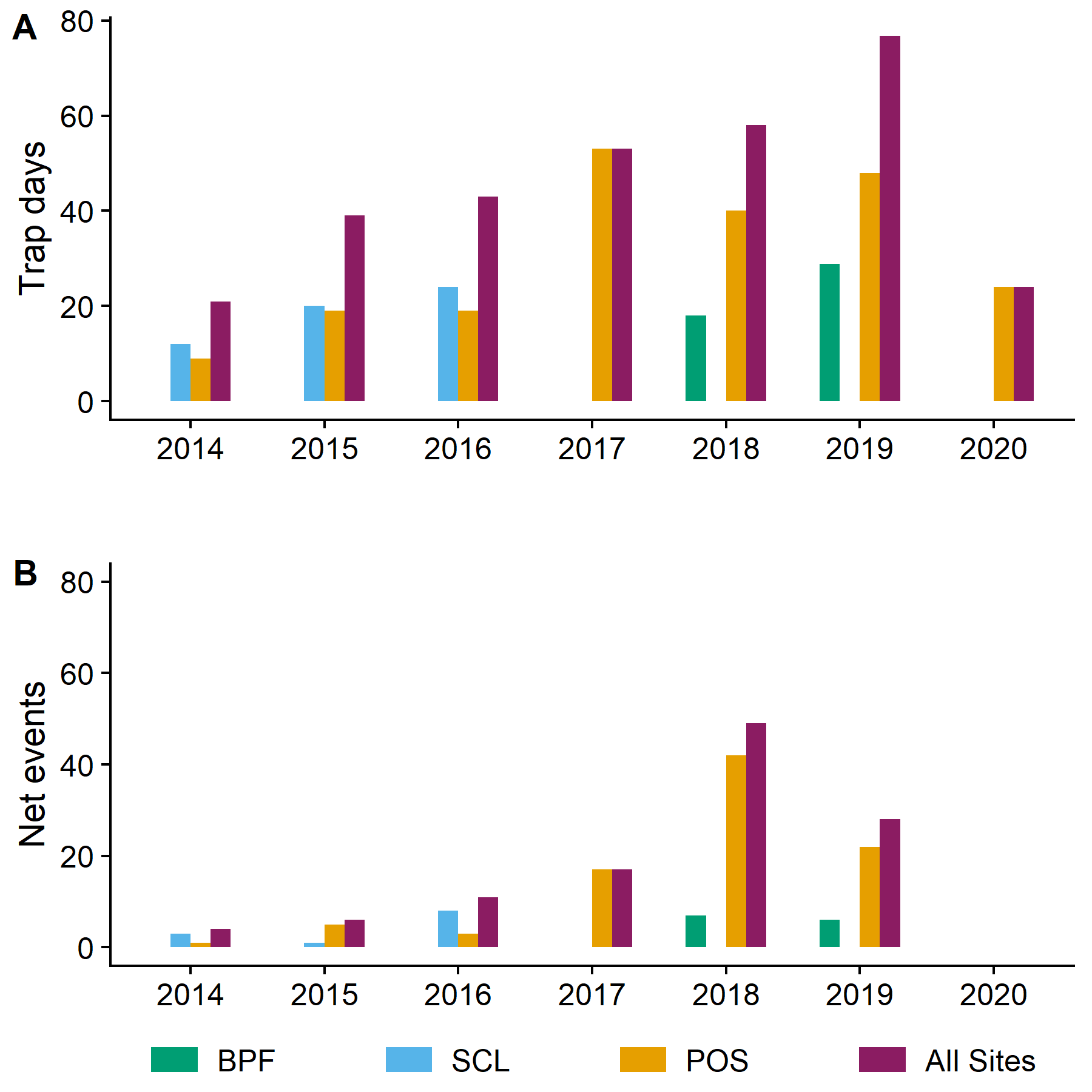
**

**Fig. S3.** Estimated genus-level seasonal distributions for parasites: (A) *Stelis*, (B) *Epeolus*, and (C) *Triepeolus*. Beneath each parasite genera are presumed host genera. Sample sizes on the right are the total records for each genus. Vertical dashed lines are 21 March, 21 June, and 21 September. These parasites have low sample sizes limiting accurate estimation.

**
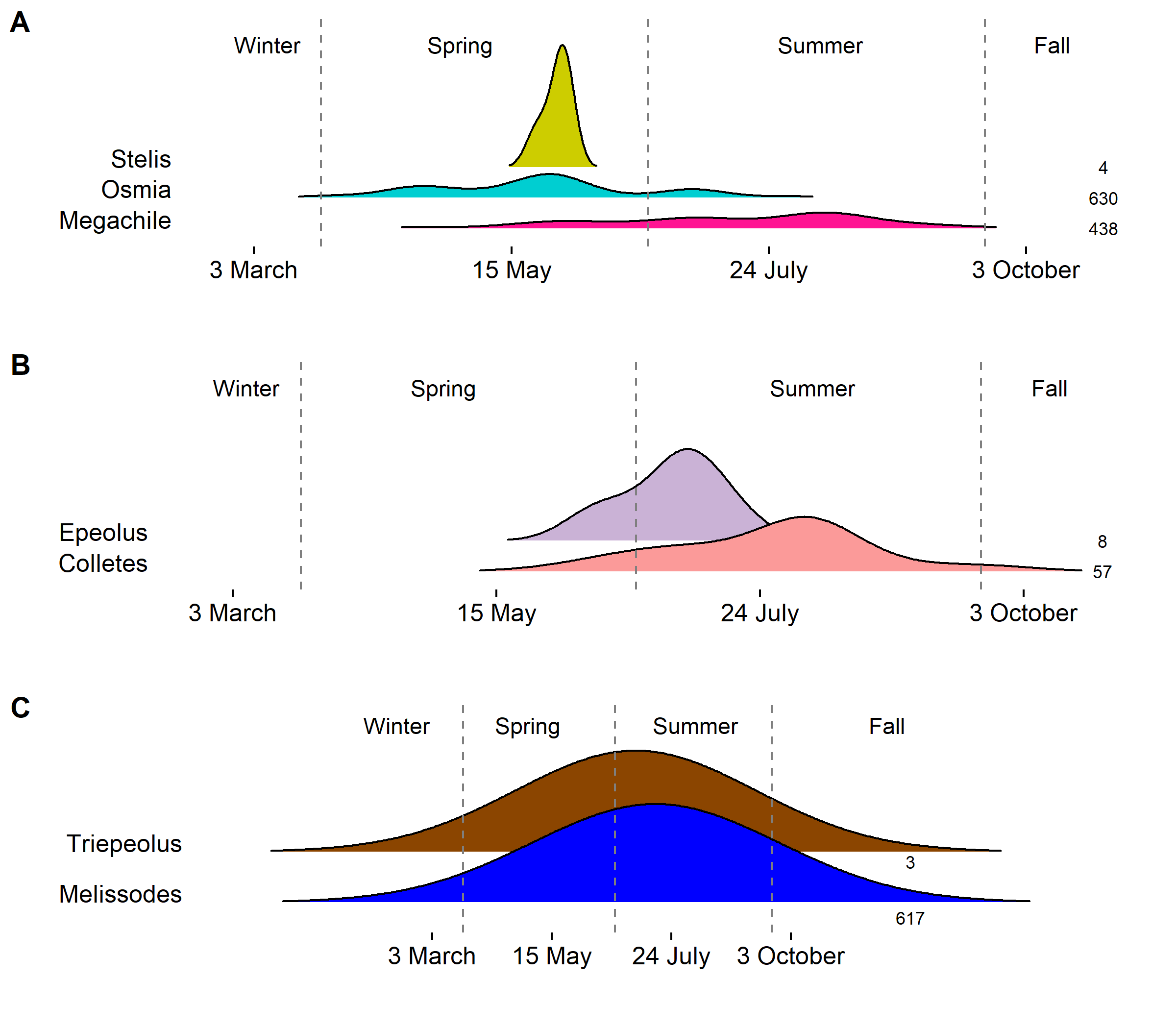
**
